# An Enhanced Differential Evolution with Elite Chaotic Local Search

**DOI:** 10.1155/2015/583759

**Published:** 2015-08-24

**Authors:** Zhaolu Guo, Haixia Huang, Changshou Deng, Xuezhi Yue, Zhijian Wu

**Affiliations:** ^1^Institute of Medical Informatics and Engineering, School of Science, Jiangxi University of Science and Technology, Ganzhou 341000, China; ^2^School of Literature and Law, Jiangxi University of Science and Technology, Ganzhou 341000, China; ^3^School of Information Science and Technology, Jiujiang University, Jiujiang 332005, China; ^4^State Key Laboratory of Software Engineering, Wuhan University, Wuhan 430072, China

## Abstract

Differential evolution (DE) is a simple yet efficient evolutionary algorithm for real-world engineering problems. However, its search ability should be further enhanced to obtain better solutions when DE is applied to solve complex optimization problems. This paper presents an enhanced differential evolution with elite chaotic local search (DEECL). In DEECL, it utilizes a chaotic search strategy based on the heuristic information from the elite individuals to promote the exploitation power. Moreover, DEECL employs a simple and effective parameter adaptation mechanism to enhance the robustness. Experiments are conducted on a set of classical test functions. The experimental results show that DEECL is very competitive on the majority of the test functions.

## 1. Introduction

Numerous problems in science and engineering can be converted into optimization problems. Therefore, it is of significance both in theory and in engineering applications to develop effective and efficient optimization algorithms for solving complex problems of science and engineering. Differential evolution (DE), proposed by Storn and Price in 1997 [1], is a simple yet effective global optimization algorithm. According to frequently reported theoretical and experimental studies, DE has exhibited competitive performance than many other evolutionary algorithms in terms of both convergence speed and solution precision over several benchmark functions and real-life problems [2–4]. Due to its simplicity, easy implementation, and efficiency, DE has stimulated many researchers' interests since its development. Therefore, it has become a hot research topic in evolutionary computation over the past decades [5–7].

However, its search ability should be further enhanced to obtain better solutions when DE is used to solve various real-life optimization problems [2, 8, 9]. Particularly, DE may suffer from premature convergence and/or slow convergence when solving complex multimodal optimization problems. In order to improve the performance of the conventional DE, a number of DE variants have been proposed in recent decades [2, 6, 10]. Recognizing that the performance of DE depends on the control parameters, Brest et al. [11] presented a self-adaptive DE (jDE), in which both *F* and CR are created independently for each individual by an adaptive mechanism. Specifically, the new *F* is created by a random value from 0.1 to 0.9 with a probability 0.1 during the search process. Meanwhile, the new CR obtains a random value from 0.0 to 1.0 with a probability 0.1. Unlike jDE, JADE, proposed by Zhang and Sanderson [12], utilizes a distinct parameter adaptation mechanism, in which the new *F* and CR are created for each individual by a normal distribution and a Cauchy distribution, respectively. In addition, JADE learns knowledge from the recent successful *F* and CR and applies the learned knowledge for creating new *F* and CR. Identifying that both the mutation strategies and their associated control parameters can directly influence the performance of DE, Qin et al. [7] proposed a novel self-adaptive DE, SaDE, which adaptively tunes the trial vector generation strategies and their associated control parameter values by extracting knowledge from the previous search process in generating promising solutions. Mallipeddi et al. [13] introduced an improved DE with ensemble of parameters and mutation strategies (EPSDE), which employs a pool of diverse trial vector generation strategies and a pool of values for the control parameters *F* and CR. By incorporating an opposition-based learning strategy into the traditional DE for population initialization and generating new solutions, Rahnamayan et al. [14] proposed an opposition-based DE (ODE). The experimental results confirmed that the opposition-based learning strategy can improve the convergence speed and the solution accuracy of DE. Further, Wang et al. [15] improved the opposition-based learning strategy, proposed a generalized opposition-based learning strategy, and presented an enhanced DE with generalized opposition-based learning strategy (GODE). Jia et al. [16] presented an effective memetic DE algorithm, DECLS, which utilizes a chaotic local search with a shrinking strategy to improve the search ability. Experimental results indicated that the performance of the canonical DE is significantly improved by the chaotic local search. Recently, Wang et al. [17] proposed a composite DE, called CoDE, the main idea of which is to randomly combine several well studied trial vector generation strategies with a number of control parameter settings highly recommended by other researchers at each generation to create new trial vectors. Experimental results on all the CEC2005 contest test instances show that CoDE is very competitive.

Although there already exist many DE variants for solving complex optimization problems, according to the no free lunch (NFL) theory [18], the performance of DE for some benchmark functions and real-life problems should be further enhanced to obtain better solutions. Moreover, many studies have revealed that embedding local search strategy can greatly enhance the search ability of DE [14, 16, 19]. Motivated by these considerations, in order to promote the performance of DE on complex optimization problems, this study proposes an enhanced differential evolution with elite chaotic local search, called DEECL. In DEECL, we utilize a chaotic search strategy based on the heuristic information from the elite individuals to promote the exploitation power. Further, we also design a simple and effective parameter adaptation mechanism to enhance the robustness.

The rest of the paper is organized as follows. The conventional DE is introduced in Section 2.  Section 3 presents the enhanced DE. Numerical experiments are presented in Section 4 for the comparison and analysis. Finally, the paper is concluded in Section 5.

## 2. Differential Evolution

Without loss of generality, only minimization problems are considered in this study. We suppose that the objective function to be minimized is Min *f*(*X*), *X* = [*X*_1_, *X*_2_,…, *X*_*D*_], and the search space is

(1) $$\begin{array}{c} \Omega =\overset{D}{\underset{j=1}{\prod }}\left[\;{\text{LB}}_{j},{\text{UB}}_{j}\;\right], \end{array}$$

where *D* is the number of dimensions of the problem, LB_*j*_ and UB_*j*_ denote the lower and upper boundaries of the search space, respectively.

Similar to other evolutionary algorithms, DE also has a simple structure, only including three simple operators, namely, mutation, crossover, and selection operators [2]. In the initial phase, DE creates an initial population *P*(*t*) = {*X*_*i*_^*t*^}, which is randomly generated from the search space, where *X*_*i*_^*t*^ = [*X*_*i*,1_^*t*^, *X*_*i*,2_^*t*^,…, *X*_*i*,*D*_^*t*^], *i* = 1,2,…, *NP*; *NP* is the population size and *t* is the generation. After initialization, the mutation and crossover operators are performed to create the trial vectors, and then the selection operator is utilized to select the better one between the offspring individual and the parent individual for the next generation. DE performs these steps repeatedly to converge toward the global optima until the terminating criterion is reached [20]. In the following subsections, the evolutionary operators of DE will be introduced in detail.

### 2.1. Mutation Operator

In the mutation operator, a mutant vector *V*_*i*_^*t*^ is created by using a predetermined mutation strategy for each individual *X*_*i*_^*t*^, namely, target vector, in the current population [17]. DE has many mutation strategies used in its implementations, such as DE/rand/1, DE/best/1, DE/rand-to-best/1, DE/best/2 and DE/rand/2 [2]. Among these mutation strategies, DE/rand/1 is the most frequently used mutation strategy, which is expressed as follows [1]:

(2) $$\begin{array}{c} V_{i}^{t}=X_{r1}^{t}+F\times \left(\;X_{r2}^{t}-X_{r3}^{t}\;\right), \end{array}$$

where *r*1, *r*2, and *r*3 are randomly selected from the set {1,2,…, *NP*}∖{*i*}, and they are mutually different from each other. *F* is called as scaling factor, amplifying the difference vector *X*_*r*2_^*t*^ − *X*_*r*3_^*t*^.

### 2.2. Crossover Operator

Following mutation, a trial vector *U*_*i*_^*t*^ is generated by executing the crossover operator for each pair of target vector *X*_*i*_^*t*^ and its corresponding mutant vector *V*_*i*_^*t*^ [2]. Binomial crossover is the most commonly used crossover operators in current popular DE. The binomial crossover is described as follows [1]:

(3) $$\begin{array}{c} U_{i,j}^{t}=\left\{\begin{array}{cc} V_{i,j}^{t}, & \text{if }\mathrm{rand}\left(0,1\right)<\text{CR or }j==jrand\\ X_{i,j}^{t}, & \text{otherwise}, \end{array}\right. \end{array}$$

where rand⁡(0,1) is generated for each *j* and takes a value from 0.0 to 1.0 in a uniformly random manner, and CR ∈ [0,1] is the crossover probability, which limits the number of parameters inherited from the mutant vector *V*_*i*_^*t*^. The integer *j*rand is randomly chosen from the range [1, *D*], which guarantees that at least one parameter of the trial vector *U*_*i*_^*t*^ is inherited from the mutant vector *V*_*i*_^*t*^ [7].

### 2.3. Selection Operator

Like the genetic algorithm, the selection process of DE is also based on the Darwinian law of survival of the fittest. The selection process is performed in order to choose the more excellent individuals for the next generation. For minimization problems, the selection operator can be defined in the following form [1]:

(4) $$\begin{array}{c} X_{i}^{t+1}=\left\{\begin{array}{cc} U_{i}^{t}, & \text{if }f\left(U_{i}^{t}\right)\leq f\left(X_{i}^{t}\right)\\ X_{i}^{t}, & \text{otherwise}, \end{array}\right. \end{array}$$

where *f*(*X*_*i*_^*t*^) and *f*(*U*_*i*_^*t*^) indicate the fitness values of the target vector *X*_*i*_^*t*^ and its corresponding trial vector *U*_*i*_^*t*^, respectively.

### 2.4. Algorithmic Framework of DE

Based on the above elaborate introduction of the DE's operators, we present the framework of DE with DE/rand/1/bin strategy in Algorithm 1, where FES is the number of fitness evaluations, Max_FES is the maximum number of evaluations, rand(0,1) indicates a random real number in the range [0,1], randint(1, *D*) represents a random integer in the range [1, *D*], and *X*_Best_^*t*^ is the global best individual found so far.

## 3. Proposed Approach

### 3.1. Motivations

DE has been demonstrated to yield superior performance for solving various real-world optimization problems [21–23]. However, it tends to suffer from premature convergence and/or slow convergence when solving complex optimization problems [6, 24]. To enhance the performance of DE, many researchers have proposed various improved DE algorithms during the past decade [25–27]. Among the DE variations, memetic method is a promising approach to improve the performance of the traditional DE, which utilizes various local search strategies, such as chaotic search strategy [16], simplex crossover search strategy [19], and orthogonal search strategy [28], to strengthen the exploitation ability of the traditional DE and consequently accelerate the convergence speed. Among the local search strategies commonly used in memetic DE, chaotic search strategy is inspired by the chaos phenomenon in nature. Chaos is a classic nonlinear dynamical system, which is widely known as a system with the properties of ergodicity, randomicity, and sensitivity to its initial conditions [16, 29, 30]. Due to its ergodicity and randomicity, a chaotic system can randomly generate a long-time sequence which is able to traverse through every state of the system and every state is generated only once if given a long enough time period [16, 31]. Taking advantage of the well-known characteristics of the chaotic systems, researchers have proposed many chaotic search strategies for optimizing various problems [16, 32–34]. However, to the best of our knowledge, among many chaotic search strategies, they pay more attention to the characteristics of the ergodicity and randomicity of the chaotic system. Therefore, the exploration capacity can be indeed improved. However, in order to maintain a balance between exploration and exploitation, the exploitation ability of the chaotic search strategy should be further enhanced. Thus, when designing a relatively comprehensive chaotic search strategy, we should further integrate more heuristic information into the chaotic search strategy to promote its exploitation power. Generally, the elite individuals in the current population known as a promising search direction toward the optimum are the favorable source that can be employed to enhance the exploitation ability. Based on these considerations, we present an elite chaotic search strategy, which not only utilizes the characteristics of the ergodicity and randomicity of the chaotic system, but also merges the superior information of the current population into the chaotic search process.

### 3.2. Elite Chaotic Search

In many chaotic search strategies, the Logistic chaotic function is utilized to generate a chaotic sequence, which is formulated as follows [16]:

(5) K0rand⁡0,1,K0≠0.25,0.5,0.75,Kn=4.0·Kn−1·1−Kn−1,n=1,2,…,N,

where *K* ^0^ is the initial value of the chaotic system, which is randomly generated from the range [0,1], but cannot be equal to 0.25, 0.5, or 0.75. *K* ^*n*^ is the *n*th state of the chaotic system. As known, the initial state *K* ^0^ of the chaotic system is randomly produced. Due to its ergodicity and sensitivity to the initial state *K* ^0^, *K* ^*n*^ is a random long-time sequence, which can traverse through every state of the system and every state is generated only once if *N* is large enough.

In order to enhance the exploitation ability of the traditional chaotic search strategy, we integrate the heuristic information learned from the elite individuals into the chaotic search strategy to promote the exploitation power. The proposed elite chaotic search strategy is defined by

(6) $$\begin{array}{c} E_{I}^{n}=X_{I}^{t}+K^{n}\times \left(\;X_{p\text{Best}}^{t}-X_{I}^{t}\;\right), \end{array}$$

where *X*_*I*_^*t*^ is an individual to be performed the elite chaotic search, which is randomly chosen from the current population. *K* ^*n*^ is the chaotic sequence, where *n* = 1,2,…, *N*, *N* = *D*/5, and *X*_*p*Best_^*t*^ is an elite individual, which is randomly chosen from the top 100*p*% individuals in the current population with *p* = rand⁡(2.0/*NP*, 0.1).

In the proposed elite chaotic search operator, an individual *X*_*I*_^*t*^ is randomly selected from the current population to undergo the elite chaotic search strategy. After that, the initial value of the chaotic system takes a value from range [0,1.0] in a uniformly random manner. Then, an elite chaotic search procedure for individual *X*_*I*_^*t*^ is repeatedly performed until finding a better solution than individual *X*_*I*_^*t*^ or the number of iterations *n* is equal to *N*. The framework of the elite chaotic search operator is described in Algorithm 2.

### 3.3. Parameter Adaptation

Since the setting of control parameters can significantly influence the performance of DE, parameter adaptation mechanism is essential for an efficient DE [7, 11, 12]. To this end, we design a simple and effective parameter adaptation mechanism inspired by [11] into DEECL. In DEECL, each individual is independently associated with its own mutation factor *F*_*i*_^*t*^ and crossover probability CR_*i*_^*t*^. For individual *i*, its control parameters *F*_*i*_^*t*^ and CR_*i*_^*t*^ are initialized to 0.5 and 0.9, respectively. Generally, a normal distribution with mean value 0.5 and standard deviation 0.3 is a promising adaptive approach for the mutation factor of DE [7], whereas Cauchy distribution is more favorable to diversify the mutation factors and thus avoid premature convergence [12]. Based on these considerations, at each generation, the new mutation factor NF_*i*_^*t*^ associated with individual *i* is generated by a Cauchy distribution random real number with location parameter 0.5 and scale parameter 0.3 with probability 0.1. Additionally, following the suggestions in [11], the new crossover probability NCR_*i*_^*t*^ associated with individual *i* acquires a random value from 0.0 to 1.0 with probability 0.1. Mathematically, the new control parameters NF_*i*_^*t*^ and NCR_*i*_^*t*^ associated with individual *i* for generating its corresponding trial vector *U*_*i*_^*t*^ are obtained by

(7) NFitrandc0.5,0.3,if  rand⁡0,1<0.1Fit,otherwise,NCRit=rand⁡0,1,if  rand⁡0,1<0.1CRit,otherwise,

where randc(0.5,0.3) is a Cauchy distribution random real number with location parameter 0.5 and scale parameter 0.3 and rand⁡(0,1) is a uniformly random number within the range [0,1]. After obtaining the new control parameters NF_*i*_^*t*^ and NCR_*i*_^*t*^, the corresponding trial vector *U*_*i*_^*t*^ are created by using the new control parameters NF_*i*_^*t*^ and NCR_*i*_^*t*^. It is widely acknowledged that better control parameter values tend to produce better individuals that have a greater chance to survive and thus these values should be propagated to the next generations [12]. Therefore, in the selection step, the control parameters *F*_*i*_^*t*+1^ and CR_*i*_^*t*+1^ associated with individual *i* for the next generation are updated by

(8) Fit+1NFit,if  fUit<fXitFit,otherwise,CRit+1=NCRit,if  fUit<fXitCRit,otherwise.

From the above designed parameter adaptation mechanism, we can infer that the better control parameters of DEECL can be propagated to the next generations. Therefore, the control parameters of DEECL can be adaptively tuned according to the feedback from the search process.

## 4. Numerical Experiments

### 4.1. Experimental Setup

In order to assess the performance of the proposed DEECL, we use 13 classical test functions (*f*1–*f*13) that are widely used in the evolutionary computation community [8, 12, 35] to verify the effectiveness of the proposed DEECL. We describe these test functions in Table 1. Among these test functions [35], *f*1–*f*4 are continuous unimodal functions. *f*5 is the Rosenbrock function which is unimodal for *D* = 2 and 3; however, it may have multiple minima in high dimension cases [36]. *f*6 is a discontinuous step function, and *f*7 is a noisy function. *f*8–*f*13 are multimodal functions and they exist many local minima [35].

In all experiments, we set the number of dimensions *D* to 30 for all these test functions. We carry out 30 independent runs for each algorithm and each test function with 150,000 function evaluations (FES) as the termination criterion. Moreover, we record the average and standard deviation of the function error value (*f*(*x*) − *f*(*x* ^*∗*^)) for estimating the performance of the algorithms, as recommended by [17], where *x* is the best solution gained by the algorithm in a run and *x* ^*∗*^ is the global optimum of the test function.

### 4.2. Benefit of the Two Components

There are two important components in the proposed DEECL: the proposed elite chaotic search strategy and the designed parameter adaptation mechanism. Accordingly, it is interesting to recognize the benefit of the two components of the proposed DEECL. For this purpose, we conduct experiments to compare the proposed DEECL with the traditional DE with DE/rand/1 strategy and two variants of DEECL, namely, DE with the proposed elite chaotic search strategy (DEwEC) and DE with the designed parameter adaptation mechanism (DEwPA). In the experiments, we set the population size of all the algorithms to 100. For the other parameters of DE and DEwEC, we set *F* = 0.5 and CR = 0.9, following the suggestions in [11].

We present the experimental results of the above mentioned algorithms in Table 2. The best results among the four algorithms are highlighted in* boldface*. “Mean Error” and “Std Dev” indicate the mean and standard deviation of the function error values achieved in 30 independent runs, respectively. From the results of comparison between DE and DEwEC, DEwEC performs better than DE on all test functions with the exception of *f*6. On test function *f*6, both DE and DEwEC exhibit similar performance. In total, DEwEC is better than DE on twelve test functions. The results of comparison between DE and DEwEC indicate that our introduced elite chaotic search strategy is effective to enhance the performance of the traditional DE.

From the comparison of DE with DEwPA, DEwPA surpasses DE on all test functions except for *f*5 and *f*6. On test function *f*6, both DE and DEwPA demonstrate similar performance, whereas DE is better than DEwPA on test function *f*5. In summary, DEwPA outperforms DE on eleven test functions. The comparison of DE with DEwPA reveals that our designed parameter adaptation mechanism is capable of improving the efficiency of the traditional DE.

**Figure 1:**
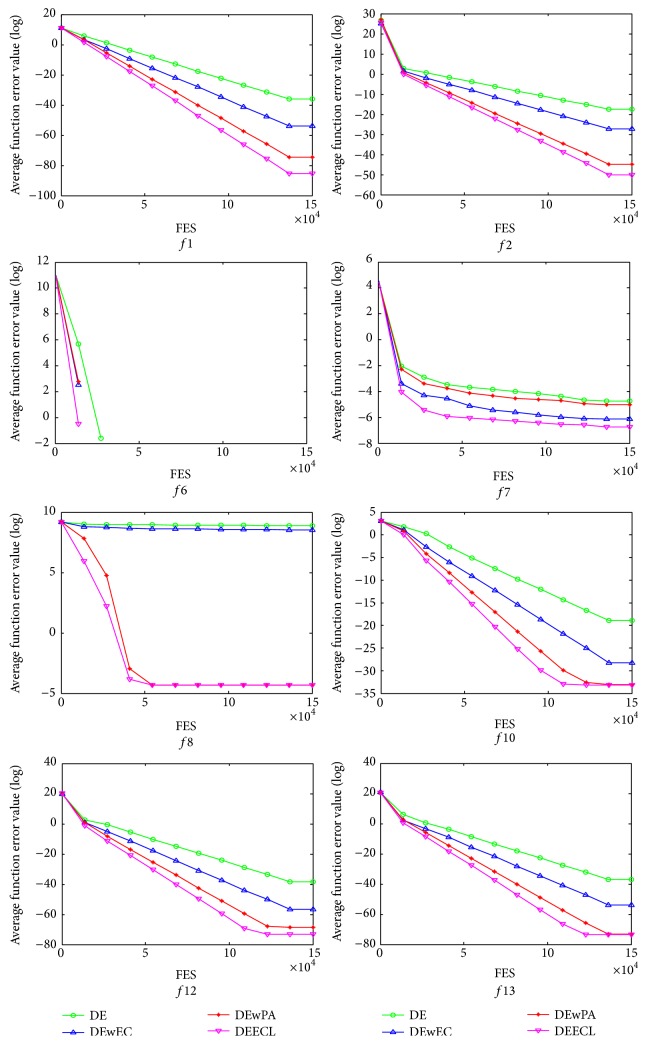
Evolution of the average function error values derived from DE, DEwEC, DEwPA, and DEECL versus the number of FES.

By incorporation of both the proposed elite chaotic search strategy and the designed parameter adaptation mechanism, DEECL achieves promising performance, which is better than other three DE algorithms on the majority of the test functions. To be specific, DEECL is better than DE, DEwEC, and DEwPA on eleven, nine, and ten test functions, respectively. DE, DEwEC, and DEwPA can outperform DEECL only on one test function. Comparison results suggest that both the introduced elite chaotic search strategy and the designed parameter adaptation mechanism demonstrate positive effect on the performance of DEECL. In addition, the comparison results confirm that the introduced elite chaotic search strategy and the designed parameter adaptation mechanism can help DE with both outperform DE with either or neither one on the majority of the test functions. Moreover, the introduced elite chaotic search strategy and the designed parameter adaptation mechanism work together to improve the performance of the traditional DE rather than contradict each other. The evolution of the average function error values derived from DE, DEwEC, DEwPA, and DEECL versus the number of FES is plotted in Figure 1 for some typical test functions. As can be seen from Figure 1, DEECL converges faster than DE, DEwEC, and DEwPA.

### 4.3. Comparison with Other DE Variants

In order to verify the effectiveness of the proposed DEECL algorithm, we compare DEECL with the traditional DE and three other DE variants, namely, jDE [11], ODE [14], and DECLS [16]. In addition, jDE is a self-adaptive DE, in which both parameters *F* and CR are generated independently for each individual by an adaptive mechanism [11]. ODE is proposed by Rahnamayan et al. [14], which incorporates the opposition-based learning strategy into the traditional DE for population initialization and creating new solutions. DECLS is an effective memetic DE algorithm [16], which utilizes the chaotic local search strategy and an adaptive parameter control approaches similar to jDE [11] to improve the search ability. In the experiments, in order to have a fair comparison, we set the population size of all the algorithms to 100. The other parameter settings of these three DE variants are the same as in their original papers.

The mean and standard deviation of the function error values achieved by each algorithm for the 13 classical test functions are presented in Table 3. For convenience of analysis, the best results among the four DE algorithms are highlighted in* boldface*. In order to gain statistically significant conclusions, we conduct two-tailed *t*-tests at the significance level of 0.05 [28, 35] on the experimental results. The summary comparison results are described in the last three rows of Table 3. “+,” “−,” and “≈” suggest that DEECL is better than, worse than, and similar to the corresponding algorithm in terms of the two-tailed *t*-tests at the significance level of 0.05, respectively.

From Table 3, we can infer that DEECL achieves the better results than all the other four algorithms on the majority of the 13 classical test functions. Specifically, DEECL is significantly better than DE, jDE, ODE, and DECLS on eleven, seven, nine, and six test functions according to the two-tailed *t*-test, respectively. In addition, DEECL is similar to DE, jDE, ODE, and DECLS on one, five, two, and five test functions, respectively. DE and jDE surpasses DEECL only on one test function. Additionally, ODE and DECLS perform better than DEECL only on two test functions.

**Figure 2:**
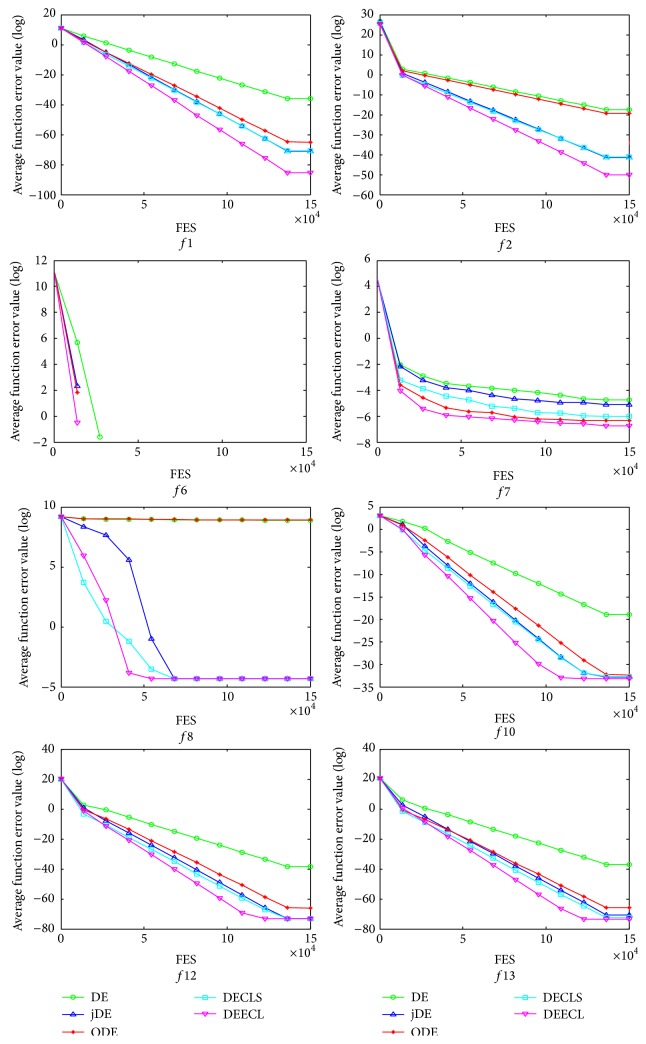
Evolution of the average function error values derived from DE, jDE, ODE, DECLS, and DEECL versus the number of FES.

Overall, DEECL performs better than the traditional DE, jDE, ODE, and DECLS on the majority of the test functions. This can be because the proposed elite chaotic search strategy learning the heuristic information from the elite individuals can promote the exploitation power, and the designed parameter adaptation mechanism can enhance the robustness. The evolution of the average function error values derived from DE, jDE, ODE, DECLS, and DEECL versus the number of FES is plotted in Figure 2 for some typical test functions. It can be known from Figure 2 that DEECL converges faster than DE, jDE, ODE, and DECLS.

In order to compare the total performance of the five DE algorithms on the all 13 classical test functions, we carry out the average ranking of Friedman test on the experimental results following the suggestions in [37–39].  Table 4 presents the average ranking of the five DE algorithms on the all 13 classical test functions. We can sort these five DE algorithms by the average ranking into the following order: DEECL, DECLS, jDE, ODE, and DE. Therefore, DEECL obtains the best average ranking, and its total performance is better than that of the other four algorithms on the all 13 test instances.

## 5. Conclusions

DE is a popular evolutionary algorithm for the continuous global optimization problems, which has a simple structure yet exhibits efficient performance on various real-world engineering problems. However, according to the no free lunch (NFL) theory, the performance of DE should be further enhanced to obtain better solutions in some cases. In this paper, we propose an enhanced differential evolution with elite chaotic local search, called DEECL, which uses a chaotic search strategy based on the heuristic information from the elite individuals to promote the exploitation power and employs a simple and effective parameter adaptation mechanism to enhance the robustness. In the experiments, we use 13 classical test functions that are widely used in the evolutionary computation community to evaluate the performance of DEECL. The experimental results show that DEECL can outperform the conventional DE, jDE, ODE, and DECLS on the majority of the test functions.

In the future, we will apply DEECL to handle more complex optimization problems, such as high-dimensional optimization problems and multiobjective optimization problems.

## Figures and Tables

**Algorithm 1:**
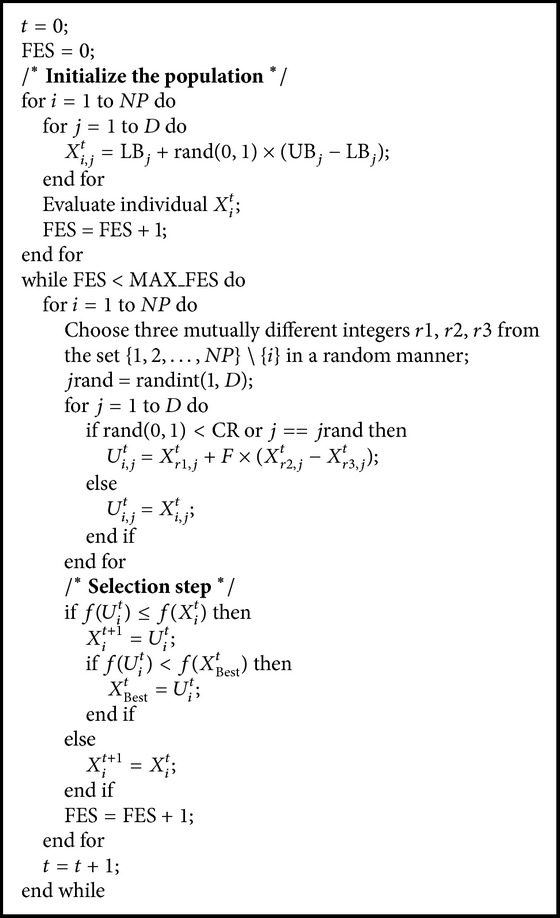
DE algorithm.

**Algorithm 2:**
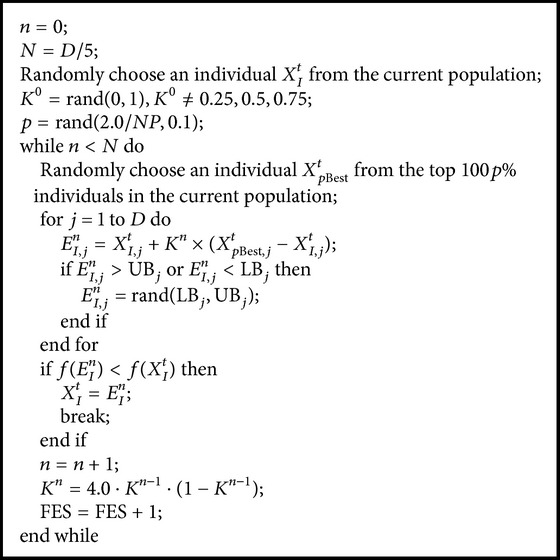
Elite chaotic search operator.

**Table 1:** The 13 classical test functions.

Function	Name	Initial range	*f*_min⁡_
*f*1	Sphere Problem	[−100,100]^*D*^	0
*f*2	Schwefel's Problem 2.22	[−10,10]^*D*^	0
*f*3	Schwefel's Problem 1.2	[−100,100]^*D*^	0
*f*4	Schwefel's Problem 2.21	[−100,100]^*D*^	0
*f*5	Rosenbrock's Function	[−30,30]^*D*^	0
*f*6	Step Function	[−100,100]^*D*^	0
*f*7	Quartic Function with Noise	[−1.28,1.28]^*D*^	0
*f*8	Schwefel's Problem 2.26	[−500,500]^*D*^	0
*f*9	Rastrigin's Function	[−5.12,5.12]^*D*^	0
*f*10	Ackley's Function	[−32,32]^*D*^	0
*f*11	Griewank Function	[−600,600]^*D*^	0
*f*12	Penalized Function 1	[−50,50]^*D*^	0
*f*13	Penalized Function 2	[−50,50]^*D*^	0

**Table 2:** Experimental results of DE, DEwEC, DEwPA, and DEECL over 30 independent runs for the 13 test functions.

Function	DE	DEwEC	DEwPA	DEECL
	Mean ± Std Dev	Mean ± Std Dev	Mean ± Std Dev	Mean ± Std Dev
*f*1	2.23*E* − 16 ± 2.50*E* − 16	4.38*E* − 24 ± 3.95*E* − 24	4.14*E* − 33 ± 4.76*E* − 33	6.89**E** − 38 ± 6.06**E** − 38
*f*2	2.86*E* − 08 ± 1.26*E* − 08	1.63*E* − 12 ± 6.75*E* − 13	3.58*E* − 20 ± 2.80*E* − 20	1.74**E** − 22 ± 1.21**E** − 22
*f*3	1.88*E* − 01 ± 6.12*E* − 02	9.12*E* − 02 ± 1.03*E* − 02	5.25*E* − 02 ± 5.94*E* − 02	2.42**E** − 02 ± 3.44**E** − 02
*f*4	1.70*E* − 01 ± 2.13*E* − 01	1.51*E* − 02 ± 2.10*E* − 02	3.23*E* − 04 ± 1.55*E* − 04	4.06**E** − 05 ± 3.05**E** − 05
*f*5	1.39*E* + 01 ± 8.74*E* − 01	1.27**E** + 01 ± 5.12**E** + 01	2.46*E* + 01 ± 1.58*E* + 01	2.95*E* + 01 ± 2.21*E* + 01
*f*6	0.00**E** + 00 ± 0.00**E** + 00	0.00**E** + 00 ± 0.00**E** + 00	0.00**E** + 00 ± 0.00**E** + 00	0.00**E** + 00 ± 0.00**E** + 00
*f*7	8.82*E* − 03 ± 2.61*E* − 03	2.15*E* − 03 ± 8.72*E* − 04	6.52*E* − 03 ± 1.59*E* − 03	1.17**E** − 03 ± 6.52**E** − 04
*f*8	7.31*E* + 03 ± 3.75*E* + 02	5.04*E* + 03 ± 3.25*E* + 02	1.53*E* − 02 ± 1.82*E* − 12	1.34**E** − 02 ± 1.19**E** − 12
*f*9	1.77*E* + 02 ± 1.10*E* + 01	0.00**E** + 00 ± 0.00**E** + 00	0.00**E** + 00 ± 0.00**E** + 00	0.00**E** + 00 ± 0.00**E** + 00
*f*10	5.93*E* − 09 ± 3.10*E* − 09	5.22*E* − 13 ± 1.76*E* − 13	4.35*E* − 15 ± 1.07*E* − 15	4.00**E** − 15 ± 0.00**E** + 00
*f*11	6.33*E* − 16 ± 1.16*E* − 15	0.00**E** + 00 ± 0.00**E** + 00	0.00**E** + 00 ± 0.00**E** + 00	0.00**E** + 00 ± 0.00**E** + 00
*f*12	2.20*E* − 17 ± 1.81*E* − 17	2.21*E* − 25 ± 1.38*E* − 25	1.61*E* − 30 ± 7.69*E* − 48	1.57**E** − 32 ± 2.74**E** − 48
*f*13	8.26*E* − 17 ± 3.59*E* − 17	4.23*E* − 24 ± 6.14*E* − 24	1.46*E* − 32 ± 2.02*E* − 33	1.36**E** − 32 ± 3.70**E** − 34

**Table 3:** Experimental results of DE, jDE, ODE, DECLS, and DEECL over 30 independent runs for the 13 test functions.

Function	DE	jDE	ODE	DECLS	DEECL
	Mean ± Std Dev	Mean ± Std Dev	Mean ± Std Dev	Mean ± Std Dev	Mean ± Std Dev
*f*1	2.23*E* − 16 ± 2.50*E* − 16+	1.51*E* − 31 ± 1.82*E* − 31+	6.20*E* − 29 ± 3.92*E* − 29+	2.01*E* − 31 ± 2.22*E* − 31+	6.89**E** − 38 ± 6.06**E** − 38
*f*2	2.86*E* − 08 ± 1.26*E* − 08+	9.13*E* − 19 ± 3.70*E* − 19+	4.31*E* − 09 ± 2.61*E* − 09+	1.46*E* − 18 ± 9.36*E* − 19+	1.74**E** − 22 ± 1.21**E** − 22
*f*3	1.88*E* − 01 ± 6.12*E* − 02+	1.85*E* − 02 ± 6.45*E* − 03**≈**	1.45*E* − 01 ± 1.17*E* − 01+	5.39**E** − 04 ± 5.78**E** − 04−	2.42*E* − 02 ± 3.44*E* − 02
*f*4	1.70*E* − 01 ± 2.13*E* − 01+	3.46*E* − 04 ± 1.23*E* − 04+	1.14**E** − 07 ± 3.43**E** − 07−	3.31*E* − 05 ± 2.00*E* − 05**≈**	4.06*E* − 05 ± 3.05*E* − 05
*f*5	1.39*E* + 01 ± 8.74*E* − 01−	1.87*E* + 01 ± 5.47*E* − 01−	2.29*E* + 01 ± 1.28*E* + 00−	5.50**E** − 05 ± 1.45**E** − 04−	2.95*E* + 01 ± 2.21*E* + 01
*f*6	0.00**E** + 00 ± 0.00**E** + 00**≈**	0.00**E** + 00 ± 0.00**E** + 00**≈**	0.00**E** + 00 ± 0.00**E** + 00**≈**	0.00**E** + 00 ± 0.00**E** + 00**≈**	0.00**E** + 00 ± 0.00**E** + 00
*f*7	8.82*E* − 03 ± 2.61*E* − 03+	5.89*E* − 03 ± 1.45*E* − 03+	1.78*E* − 03 ± 6.21*E* − 04+	2.45*E* − 03 ± 2.36*E* − 03+	1.17**E** − 03 ± 6.52**E** − 04
*f*8	7.31*E* + 03 ± 3.75*E* + 02+	1.34**E** − 02 ± 1.82**E** − 12**≈**	7.51*E* + 03 ± 2.36*E* + 02+	1.34**E** − 02 ± 1.82**E** − 12**≈**	1.34**E** − 02 ± 1.19**E** − 12
*f*9	1.77*E* + 02 ± 1.10*E* + 01+	0.00**E** + 00 ± 0.00**E** + 00**≈**	7.83*E* + 01 ± 2.21*E* + 01+	0.00**E** + 00 ± 0.00**E** + 00**≈**	0.00**E** + 00 ± 0.00**E** + 00
*f*10	5.93*E* − 09 ± 3.10*E* − 09+	5.42*E* − 15 ± 1.74*E* − 15+	8.97*E* − 15 ± 1.74*E* − 15+	6.48*E* − 15 ± 1.63*E* − 15+	4.00**E** − 15 ± 0.00**E** + 00
*f*11	6.33*E* − 16 ± 1.16*E* − 15+	0.00**E** + 00 ± 0.00**E** + 00**≈**	0.00**E** + 00 ± 0.00**E** + 00**≈**	0.00**E** + 00 ± 0.00**E** + 00**≈**	0.00**E** + 00 ± 0.00**E** + 00
*f*12	2.20*E* − 17 ± 1.81*E* − 17+	1.97*E* − 32 ± 8.15*E* − 33+	2.23*E* − 29 ± 2.32*E* − 29+	1.64*E* − 32 ± 1.55*E* − 33+	1.57**E** − 32 ± 2.74**E** − 48
*f*13	8.26*E* − 17 ± 3.59*E* − 17+	2.09*E* − 31 ± 2.93*E* − 31+	2.57*E* − 29 ± 3.07*E* − 29+	3.25*E* − 32 ± 2.37*E* − 32+	1.36**E** − 32 ± 3.70**E** − 34

−	1	1	2	2	
+	11	7	9	6	
≈	1	5	2	5	

**Table 4:** Average rankings of the five algorithms for the 13 test functions achieved by Friedman test.

Algorithm	Ranking
DEECL	**2.04**
DECLS	2.27
jDE	2.65
ODE	3.50
DE	4.54
